# C-Phycocyanin-Derived Peptide-Conjugated, Chemically Crosslinked Chitosan/Carrageenan Hydrogels for Myoblast Cultivation in Reduced Serum Conditions

**DOI:** 10.3390/gels12080720

**Published:** 2026-08-13

**Authors:** Ljubodrag Aleksić, Vladimir Pavlović, Marija S. Nikolić, Aleksandar Ivanov, Nikola Gligorijević, Simeon Minić

**Affiliations:** 1 Centre of Excellence for Molecular Food Sciences & Department of Biochemistry, Faculty of Chemistry University of Belgrade, 11158 Belgrade, Serbia; aleksic@chem.bg.ac.rs (L.A.);; 2 Department of Mathematics and Physics, Faculty of Agriculture, University of Belgrade, 11080 Belgrade, Serbia; 3 Department of General and Inorganic Chemistry, Faculty of Technology and Metallurgy, University of Belgrade, 11000 Belgrade, Serbia; 4 Department of Chemistry, Institute of Chemistry, Technology, and Metallurgy, University of Belgrade, 11000 Belgrade, Serbia

**Keywords:** hydrogel, chitosan, carrageenan, peptides, myoblasts, serum

## Abstract

Fetal bovine serum (FBS) is commonly added to cell culture media, but for cultivated meat production to become truly sustainable, ethical and affordable, an alternative to it is necessary. The purpose of this study was to demonstrate how myoblasts could be cultivated in reduced-serum media using animal-free scaffolds. Novel chitosan/carrageenan hydrogels were developed and conjugated with peptides obtained by digestion of C-phycocyanin from cyanobacteria *Arthrospira platensis*, their physico-chemical properties were assessed, and their ability to serve as scaffolds for myoblast cultivation in reduced-serum media was examined. These hydrogels were found to possess suitable physical characteristics to serve as scaffolds for cell culture, and it was demonstrated that their use made quail muscle clone 7 (QM7) cell cultivation possible in reduced-FBS media. These hydrogels, therefore, are a promising candidate for use as scaffolds in future cultivated meat production studies.

## 1. Introduction

The growth of the global population, increasing consumer concern for animal welfare, and environmental concerns are driving research into alternatives to traditional meat production [1]. Conventional animal agriculture struggles to keep up with demand and has a massive negative impact on the environment because of the energy, land, water use, and greenhouse gas emissions associated with it. A more sustainable and animal-friendly alternative is therefore necessary [2]. Cultivated meat (CM) is one of the sustainable alternatives to ‘traditional’ meat currently being explored [1].

The ‘tissue-based’ approach to CM production involves culturing muscle cells on edible scaffolds [1]. Porous scaffolds such as hydrogels [2], three-dimensional macromolecular networks formed by cross-linked hydrophilic polymer chains that are highly swollen in water (but do not dissolve in it) [3], can be used for cell culture, as they can mimic the structure of the extracellular matrix (ECM) and enable nutrient and gas exchange [2]. However, scaffolds used in CM production are often based on animal-derived biomacromolecules [4]; since a key aim of cultivated meat production is to preclude the slaughter of animals, animal-derived biomaterials should be avoided [5].

Algal biopolymers have been used in tissue engineering [5] and as scaffolds for cultured meat [6]. Algal polysaccharides that intrinsically form hydrogels, whose main source is seaweed, include alginate, carrageenan, starch, agarose and cellulose [3]. Carrageenan and chitosan are among the polysaccharides used in the food industry that have gelling abilities [7].

Carrageenan is a set of structurally related sulfated polysaccharides, including κ-carrageenan, the backbone of which is made of β-D-galactose and 3,6-anhydro-α-L-galactose. Widely cultivated seaweed genera *Euchema*/*Kappaphycus* and *Gracilaria* are mainly used for carrageenan and agar extraction [3]. Carrageenans are approved by the Food and Drug Administration and are Generally Regarded as Safe; they are widely used as gelling, emulsifying and thickening agents in food products [8]. Κ-carrageenan notably offers tunability in terms of gel texture and mechanical properties but often suffers from limitations such as brittleness and low flexibility: a well-established strategy to overcome these is blending with other hydrocolloids, enhancing properties including structural stability and water retention [8].

Chitosan is a non-toxic, biocompatible, and sterilizable [9] chitin derivative made of glucosamine and *N*-acetyl glucosamine residues covalently linked together in polysaccharide chains. It is found in diatoms, brown and green algae, among other organisms, though it is currently mainly derived from crustaceans for industrial applications. Chitosan’s reactive amino and hydroxyl groups confer many functional properties, making it a versatile material used in the food industry for food packaging, edible coatings, emulsification, thickening, stabilization and food preservation in addition to other applications [10].

Electrostatic interactions between positively charged chitosan and negatively charged carrageenan may lead to the formation of a so-called polyelectrolyte complex (PEC) [11].

Genipin, a biocompatible iridoid mainly extracted from *Genipa americana* and *Gardenia jasminoides* fruits [12], is a bi-functional crosslinking agent that reacts, for example, with chitosan and/or protein molecules *via* their primary amine groups to produce non-cytotoxic blue hydrogels [9].

As mentioned previously, blending different hydrocolloids often enhances various gel characteristics [8] such as stiffness, viscoelasticity, porosity, topography and ligand presentation, which can influence cell adhesion, migration, proliferation and differentiation [13].

Most non-animal-derived scaffolds support limited cell adhesion [14], but materials’ ability to support cell adhesion can be improved through (bio)chemical modification, such as peptide immobilization [15]. The most commonly used peptide motif used for these purposes is the RGD tripeptide sequence found in ECM proteins such as fibronectin, where it acts as a binding site for transmembrane integrins [16], promoting cell adhesion and migration [5].

Fetal bovine serum (FBS) is frequently added to cell growth media as it notably contains, among other components, cell attachment factors. However, since it is an animal-derived product [17] it can raise serious ethical and sustainability concerns [18], its use is inconsistent with the goals of cultivated meat production [17] and alternatives to it should be developed [18,19].

C-phycocyanin is the most abundant protein in cyanobacteria *Arthrospira platensis* (Spirulina) [20]. The β subunit of C-phycocyanin contains several chymotrypsin cleavage sites, as well as an RGE sequence that is not typically affected by chymotrypsin [21,22], meaning that a chymotrypsin digestion of *Arthrospira platensis* extract likely yields RGE sequence-containing peptides. These peptides potentially enhance the cell adhesive properties of hydrogels in a manner similar to RGD sequence-containing peptides, allowing cells to colonize a hydrogel in cell growth media containing a significantly lower concentration of FBS than is typically used. In fact, RGE sequence-containing peptides have been demonstrated to have a similar pro-adhesive effect to RGD sequence-containing peptides in a study by Mantz et al. [23].

In the present study, combined chitosan/carrageenan hydrogels were prepared to obtain a mechanically sturdy scaffold to be assessed as a platform for the production of cultivated meat. A subset of these hydrogels was prepared in the presence of a chymotrypsin digest of an *Arthrospira platensis* extract to yield hydrogels potentially enriched with covalently attached peptides, containing the RGE amino acid sequence speculated to enhance cell adhesion in a manner similar to the RGD sequence. The techno-functional properties of these gels (rheology, water retention capacity, water uptake capacity) were determined, scanning electron microscopy and Fourier-transform infrared spectroscopy (FTIR) were performed, and the ability of quail muscle clone 7 (QM7) myoblasts to grow on these hydrogels was demonstrated, confirming the suitability of these hydrogels as platforms for long-term myoblast cultivation. Moreover, it was demonstrated that chitosan/carrageenan hydrogels potentially containing immobilized peptides from *Arthrospira platensis* allowed for successful QM7 cell cultivation in reduced-FBS cell growth media.

## 2. Results and Discussion

**Figure 1:**
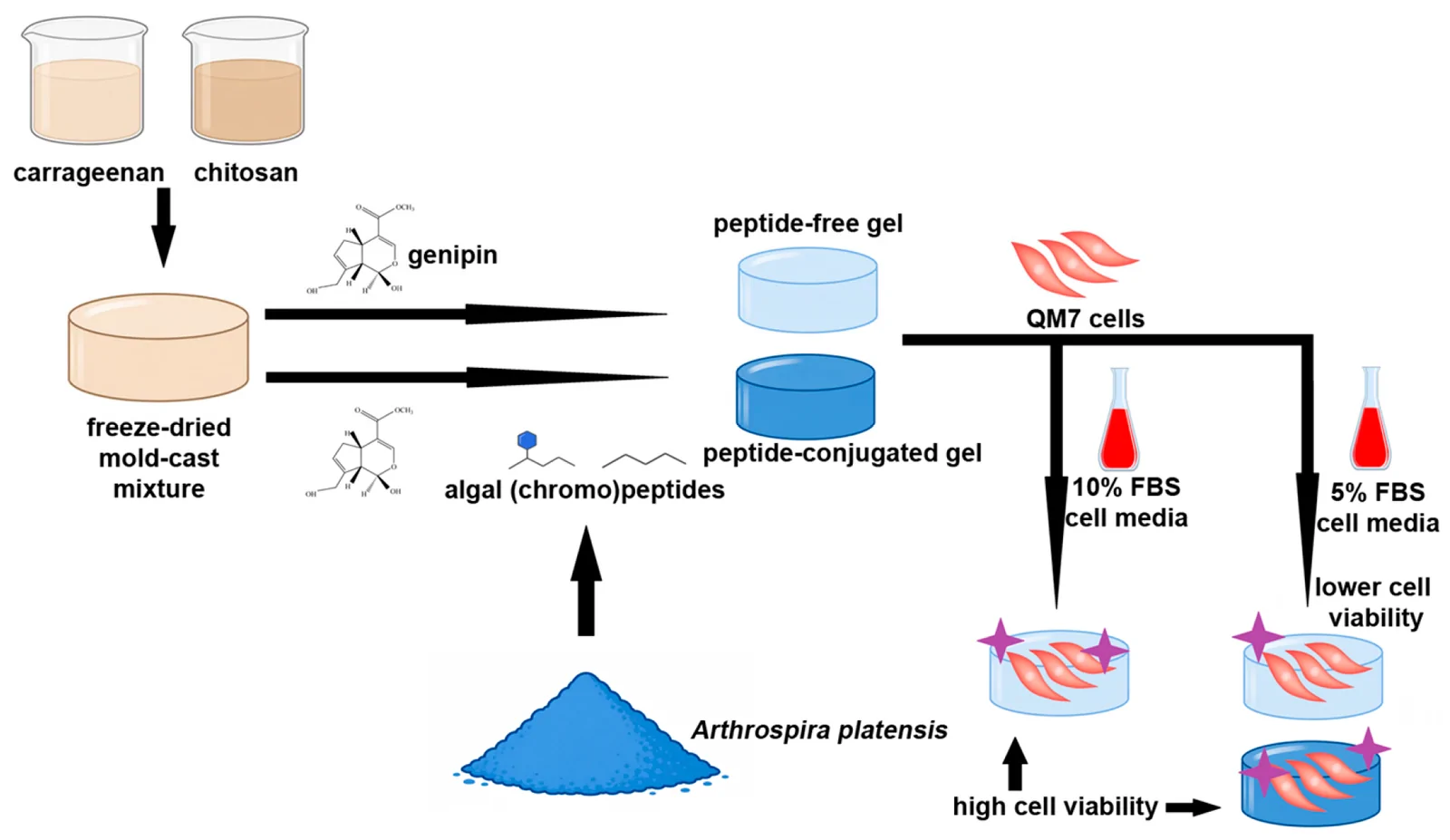
Outline of the main part of the present study.

For the sake of clarity and ease of readers’ comprehension, an outline of the most important aspects and aims of the present paper is shown in Figure 1 prior to all experimental results.

The first step depicted in Figure 1 is the fabrication of a chitosan/carrageenan polyelectrolyte complex using a chitosan solution and a carrageenan suspension of equal concentrations, which is subsequently freeze-dried. In the next step, freeze-dried polyelectrolyte complexes are cross-linked using genipin, and some of them are simultaneously modified using *A. platensis*-derived peptides to yield peptide-free and potentially peptide-conjugated hydrogels. In the final step depicted, these are used to cultivate QM7 cells, where the ability of non-conjugated gels to support QM7 growth in non-reduced serum and reduced serum conditions is compared to the ability of conjugated gels to do the same in reduced-serum conditions.

### 2.1. Hydrogel Preparation and Scanning Electron Microscopy

**Figure 2:**
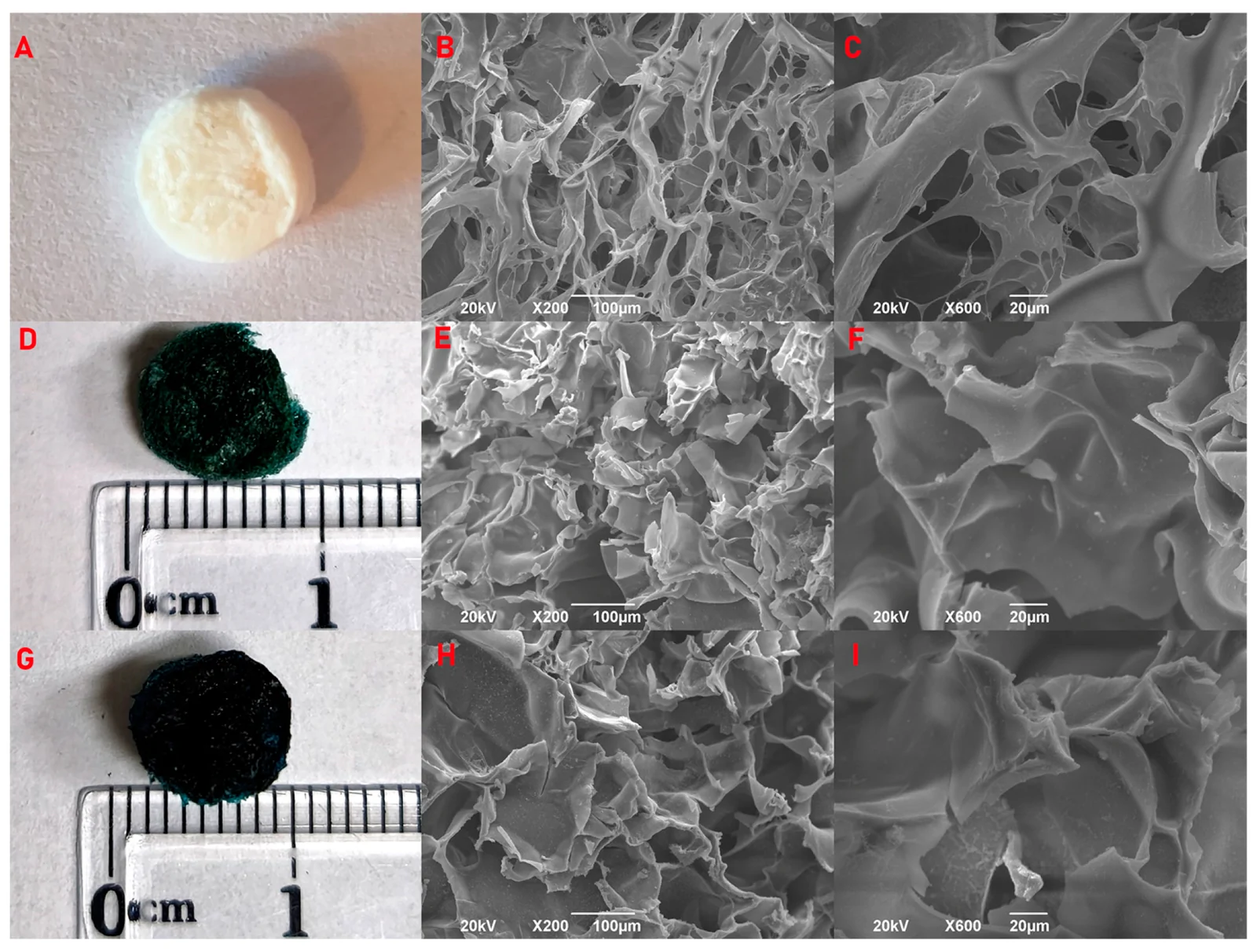
Photographs of the freeze-dried chitosan/carrageenan mixture used to prepare hydrogels (**A**) and SEM images of this mixture (**B**,**C**); a photograph of a peptide-free genipin-crosslinked chitosan/carrageenan hydrogel (**D**) and SEM images of this gel (**E**,**F**); a photograph of a potentially peptide-conjugated genipin-crosslinked chitosan/carrageenan hydrogel (**G**) and SEM images of this gel (**H**,**I**).

A freeze-dried chitosan/carrageenan mixture, a peptide-free chitosan/carrageenan hydrogel, a potentially peptide-enriched chitosan/carrageenan hydrogel photographs and their surfaces examined using scanning electron microscopy are shown in Figure 2.

Immediately noticeable is the difference in color between the starting material (non-crosslinked chitosan/carrageenan mixture and the end result of genipin-induced cross-linking. As mentioned previously, the appearance of this blue color is expected following successful genipin-mediated cross-linking [9]. Further, a difference in color can also be observed between the peptide-free chitosan/carrageenan gel and the potentially peptide-enriched one, with the potentially peptide-enriched gel being noticeably darker, with a more saturated blue hue. This suggests that blue *Arthrospira platensis* chromopeptides, carrying phycocyanobilin, have likely successfully been covalently linked to chitosan, along with RGE sequence-containing peptides.

As can be seen from Figure S1, after 24 h incubation, there is a significantly lower amount (more than four times) of retained peptides by gels in the absence of genipin compared to the genipin-induced crosslinked gels. This suggests genipin-induced covalent peptide immobilization.

SEM images of gels are presented in Figure 2, and it can be seen that the addition of genipin significantly decreases the number and size of pores, to the point that they could not be clearly observed in the images captured by SEM. This observation is supported by the results of the ImageJ (version 1.54g) analysis—the pore count of the non-crosslinked sample in the ×200 magnification image was found to be 154, the average pore size was found to be 121.5 ± 16.0 μm^2^, and the percentage of the image area taken up by the pores was found to be 6.7%, while no pores were clearly visible in the SEM images of the cross-linked samples. It is important to acknowledge that the ImageJ analysis used to estimate pore size and number in the present study has limitations. Firstly, as Gomber et al. [24] have pointed out, it is rash to draw firm conclusions about pore size based on SEM alone, and secondly, it is important to clarify that the values obtained *via* ImageJ analysis are not to be taken as absolutely, but only as indicative of a trend of chemical crosslinking leading to fewer and smaller pores.

Indeed, this trend has been reported in the past, by, for example, Stefanov et al. [25]: thiolated chitosan hydrogels for wound dressing were fabricated using chicoric acid as a cross-linker, and it was found that increasing chicoric acid concentration from 0.24 mM to 0.73 mM leads to a decrease in pore diameter from 22.0 ± 8.5 μm to 6.5 ± 2.2 μm in gels made with a thiolated chitosan called TCS2-5 by the authors. Further, Holmes and Stellwagen [26] examined the dependence of pore size of polyacrylamide gels on the concentration of *N*,*N*′-methylenebisacrylamide, which ranged from 0.5 to 10% (*w*/*w*) with respect to total acrylamide. It was observed that for any given concentration of total acrylamide, the smallest pore radii were found in gels made with 5 or 10% *N*,*N*′-methylenebisacrylamide, and the largest pore radii were found in gels made with 1–2% of this crosslinker. Slightly smaller pores were in gels made with 0.5%, *N*,*N*′-methylenebisacrylamide, which Holmes and Stellwagen [26] hypothesized was caused by an increase in flexibility of the matrix at very low crosslinker concentrations. Additionally, Wong, Ashton and Dodou [27] examined the hydrogel network density of polyethylene oxide hydrogels with increasing pentaerythritol tetra-acrylate (PETRA), a UV-activated cross-linker, and found that an increase in PETRA concentrations leads to a denser hydrogel network, with a decrease in mesh size.

### 2.2. Fourier-Transform Infrared Spectroscopy

**Figure 3:**
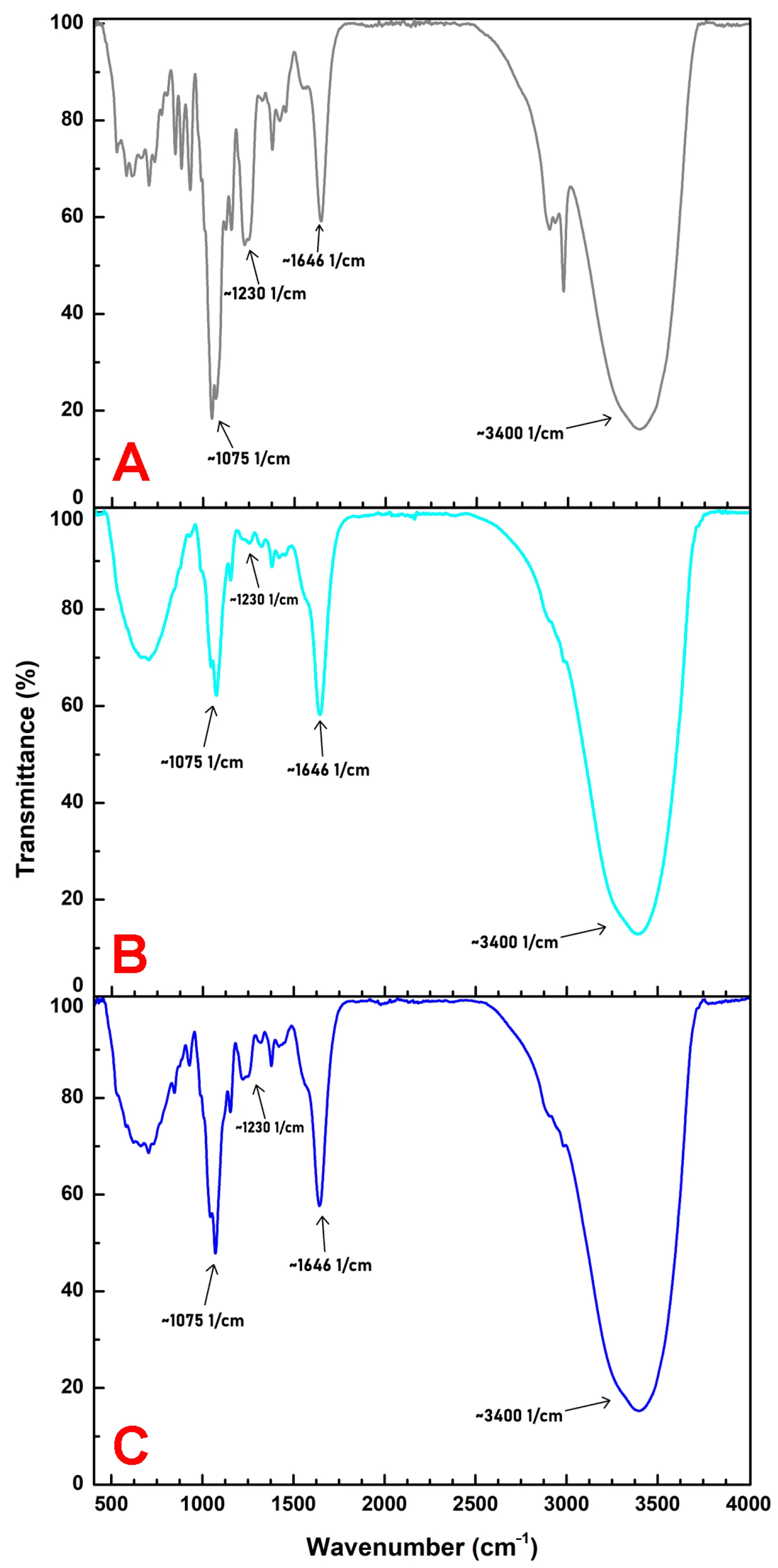
Infrared spectra of the freeze-dried chitosan/carrageenan mixture used to prepare hydrogels (**A**), a peptide-free genipin-crosslinked chitosan/carrageenan hydrogel (**B**) and a potentially peptide-conjugated genipin-crosslinked chitosan/carrageenan hydrogel (**C**).

The FTIR spectra of the freeze-dried chitosan/carrageenan hydrogels with and without potentially immobilized peptides, as well as the non-crosslinked mixture of chitosan and carrageenan used to prepare these gels are shown in Figure 3. The FTIR spectra of individual chitosan and carrageenan polymers used in this study are shown in Figure S2.

In the FTIR spectrum of chitosan (Figure S2), several prominent bands can be observed. At around 1078 cm^−1^, there is a band attributable to C-O stretching [28], specifically to CH_2_-OH and CH-OH stretching, as well as to C-N stretching [29]. Further, the amide III band reported by Deka et al. [28] at 1424 cm^−1^ can be observed at around 1410 cm^−1^, the amide II band is present around 1560 cm^−1^, and the amide I band is present around 1650 cm^−1^, originating from C=O stretching vibration. All these spectral features are due to the incomplete deacetylation of chitin during the obtaining of chitosan.

In the FTIR spectrum of carrageenan (Figure S2), there are several prominent bands. A band assigned to C-O stretching, specifically C-OH stretching vibrations, can be observed around 1075 cm^−1^ [28], which could also partly be attributed to S=O (of -O-SO_3_- moieties) asymmetric stretching, a band attributable to S=O (of -O-SO_3_- moieties) symmetric stretching is present around 1261 cm^−1^ [29], and a band around 1436 cm^−1^ may be attributed to CH_3_ and CH_2_ bending vibrations [28].

It is important to note that the spectrum of the chitosan/carrageenan mixture is different from either polysaccharide in isolation.

A prominent band at around 1646 cm^−1^ is present in the FTIR spectra of all five samples. A similar band was reported by Dacrory, Hashem, and Kamel [30] in a chitosan/carrageenan mixture at 1634 cm^−1^, which was explained as the result of a combination of a band observed at 1630 cm^−1^ in carrageenan spectra, attributable to the angular deformation of hydroxyl groups of water (in fact, H-O-H bending vibrations) bound to carrageenan chains [31], and the amide I (C=O stretching) peak of chitosan [28,32,33]. In the case of crosslinked samples, the band around 1646 cm^−1^ can be attributed to secondary amide groups and C=O stretching vibrations, present due to the incomplete deacetylation of chitosan, the amide groups resulting from genipin-induced crosslinking [34] and residual water (H-O-H bending) bound to carrageenan [31]. The broad band at around 3400 cm^−1^ present in all samples is assigned to the stretching vibration of O-H bonds belonging to hydroxyl groups of both chitosan and carrageenan [28], N-H stretching vibration in secondary amide moieties [35] in the case of chitosan and the chitosan/carrageenan polyelectrolyte complex, and in the case of crosslinked samples, possibly to the stretching vibration of O-H bonds belonging to hydroxyl groups in genipin.

Of note is the near disappearance of the amide II band at around 1560 cm^−1^, present in the spectrum of chitosan but absent in the chitosan/carrageenan samples. A similar phenomenon reported by Deka et al. [28] was attributed to chitosan-carrageenan interactions. Other prominent bands become significantly less intense following genipin-induced cross-linking: the band around 1230 cm^−1^, and that located between 1000 and 1100 cm^−1^, suggesting changes within polysaccharide network upon covalent cross-linking, with a direct involvement of sulfate groups of carrageenan.

### 2.3. Rheological Property Analysis

**Figure 4:**
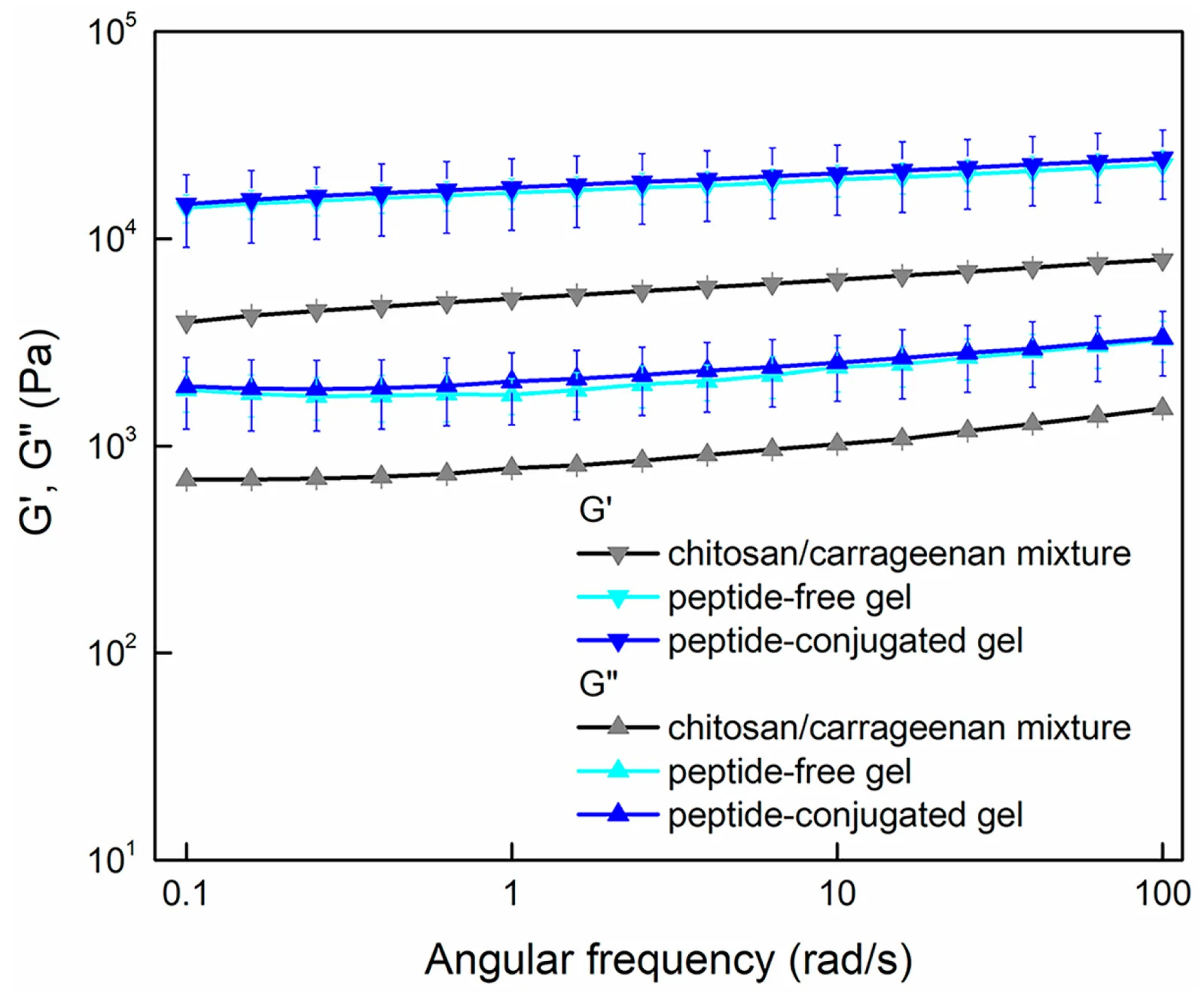
Results of the rheological property analysis: comparison of G′ and G″ of a non-crosslinked chitosan/carrageenan mixture with the G′ and G″ of a peptide-free and a potentially peptide-conjugated genipin-crosslinked chitosan/carrageenan hydrogel, all hydrated using a 50 mM phosphate buffer (pH 7.2).

The results of the analysis of the rheological properties of genipin-crosslinked hydrogels with and without potentially immobilized C-phycocyanin-derived peptides, as well as adequate control samples, are depicted in Figure 4.

All three samples exhibit solid-like behavior, as their storage moduli (G′), an indicator of the amount of energy elastically stored and released during deformation, are higher than their loss moduli (G′), a measure of how energy is irreversibly dissipated as kinetic/flow energy or heat when a deformation process of a sample is carried out (viscous behavior). Additionally, since G′ may be used to compare the mechanical strength of different samples in oscillatory rheological experiments, it can be concluded that genipin-crosslinked hydrogels, regardless of the presence of potentially immobilized peptides, have significantly higher storage moduli than chitosan/carrageenan mixtures not crosslinked with genipin. A noticeable improvement to the mechanical properties of chitosan/carrageenan hydrogels can be attributed to the genipin crosslinking effect induced on the chitosan macromolecules in the polysaccharide mixtures investigated.

Furthermore, a weak frequency dependence of the moduli was observed for all investigated gels. The storage modulus was fitted to the power law, G′(ω)~ω^n^, and the resulting scaling exponent yielded a small but non-zero value—the values of *n* were 0.097 ± 0.011 for the non-crosslinked sample, 0.067 ± 0.001 for the peptide-free gel, and 0.071 ± 0.001 for the potentially peptide-conjugated gel. A decrease in the value of *n* is indicative of the formation of a structured polymer network, although it did not reach zero. While an ideal chemically (via covalent and/or ionic bonds) crosslinked gel exhibits zero frequency dependence of its dynamic modulus [36], real gel networks can display a slight frequency dependence. In this case, the frequency dependence likely originates from structural heterogeneities, the relaxation of dangling chain ends, or network defects such as loop formation during crosslinking.

Moura, Figueiredo and Gil [37] performed similar oscillatory rheological experiments to determine the G′ and G″ of chitosan hydrogels crosslinked with different concentrations of genipin. A 1.5% chitosan solution (*w*/*v*) was used to make hydrogels, which is the same final concentration of chitosan used in the present study. Different genipin concentrations were used, including 0.1% (*w*/*v*) genipin. Samples without genipin were used as controls. An oscillatory frequency sweep experiment was performed after 12 h of crosslinking. It was found that all the hydrogels exhibited a G′ plateau in the range of 0.01–10 Hz, equivalent to ~0.06–~62 rad/s, followed by a steady increase. The rheological behavior in the range of angular frequency of 0.06–62 rad/s resembles the behavior exhibited by the chitosan/carrageenan gels used in the present study. However, the G′ values of the crosslinked chitosan gels made by Moura, Figueiredo and Gil [37] using 0.1% genipin was significantly lower than the G′ values of the chitosan/carrageenan used in this work. The degree to which this difference can be attributed to the presence of carrageenan as opposed to the time allowed for crosslinking to happen is yet to be determined.

Due to the poor mechanical properties of the non-crosslinked chitosan/carrageenan mixture, it was not used in further experiments.

### 2.4. Water Uptake and Retention Capacity

**Figure 5:**
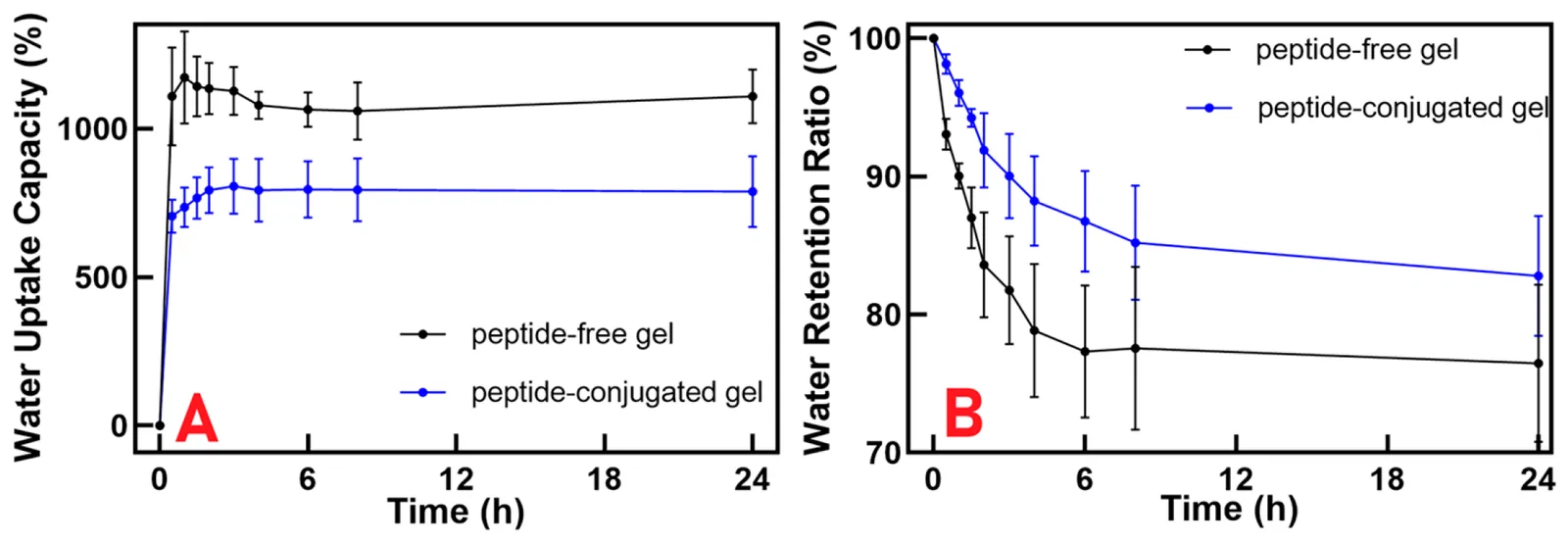
Graphs of the water uptake capacity (**A**) and water retention properties (**B**) of peptide-free and potentially C-phycocyanin-derived peptide-conjugated genipin-crosslinked chitosan/carrageenan hydrogels during a period of 24 h.

Graphs showing the water uptake and water retention capacity of chitosan/carrageenan gels with and without potentially immobilized C-phycocyanin-derived peptides are depicted in Figure 5.

After a period of 30 min, the mass of peptide-free gels whose water uptake capacity was being measured was found to be 10-fold higher than the original mass; whereas, the mass of potentially peptide-enriched gels was found to be 8-fold higher than the original mass (Figure 5A). Tubić et al. [38] includes a table that contains the water uptake capacity of various hydrogels as reported by several authors. Notably, the water uptake capacities of a chitosan hydrogel and a κ-carrageenan/locust bean gum were determined by Borzacchiello et al. [39] and Pettinelli et al. [40], respectively. Although these hydrogels were formulated and fabricated differently from those in the present study, and although different solutions were used to study their water uptake properties, it is notable that the values reported are significantly lower than those obtained for genipin-crosslinked chitosan/carrageenan hydrogels. Further, many other hydrogels were also reported to have significantly lower water uptake capacities, while only a few were reported to have comparable or higher swelling ratios by comparison with the hydrogels described in the present study; among these are the hydrogels developed by Tubić et al. [38], as well as those described in Basu, Saha and Saha [41], Slavutsky and Bertuzzi [42], and Panariello et al. [43]. Also of note is that few of the gels displayed such a high water uptake rate similar to that observed for the chitosan/carrageenan hydrogels in the present study [38].

As can be seen in Figure 5A, the water uptake ability of the non-conjugated networks was found to be superior to that exhibited by the potentially peptide-conjugated matrices: the peptide-free systems reached a maximum degree of swelling (practically, within experimental error limits a plateau was attained) after 30 min, while the potentially peptide-conjugated ones displayed a water uptake that continued to slowly increase for another hour and a half prior to reach its own plateau. The adverse effect of potentially immobilized peptides on water uptake is somewhat surprising. Ghaffari-Bohlouli et al. [44] compared the swelling capacity of hyaluronic acid-tyramine-NH_2_ hydrogels to the same gels functionalized using a VLSTSFPPW peptide and found that the swelling capacity of conjugated hydrogels was 1.5-fold higher than the swelling capacity of non-conjugated ones. Further, Zhu et al. [45] reported a similar effect-RRRFRGDK-conjugated photo-crosslinked 4-Lys-4-MA, mPEG-MA and AEMA hybrid hydrogels were found to have a slightly higher swelling ratio than non-conjugated gels, while Lin, Metters and Anseth [46] reported that the immobilization of YCWSQYLCY (di-sulfide form) up to concentrations of 500 μM had no effect on the swelling capacity of poly(ethylene glycol) hydrogels.

As it has been shown that the swelling of covalently crosslinked alginate hydrogels is strongly influenced by the hydrophilic/hydrophobic character of the crosslinking agent, as well as by the crosslinking density [47], in the case of genipin-crosslinked systems investigated in the present study, the factors mentioned above can affect, in a complex manner, the water swelling behavior of these matrices, the complexity of the process being most likely due to the influence of the presence of peptides during the crosslinking process (efficiency and overall crosslinking density) compared to the same process performed on the peptide-free chitosan/carageenan systems.

In the water retention experiment (Figure 5B), it is noticeable that peptide-free gels lose mass faster than potentially peptide-conjugated gels, as evidenced by a steeper decline in the water retention ratio, especially during the first 5 h. Further, it was found that the mass of both types of gel reached a plateau after roughly 10 h, though with a certain difference between the two groups, with potentially peptide-conjugated gels retaining more water. It is possible that while hydrogel surface-bound hydrophobic peptides decrease the water uptake rate and the equilibrium swelling ratio, they could simultaneously diminish the rate of water release/removal from these swollen hydrogels. These findings require further investigation to be clarified fully for a better understanding and a rational comparative analysis of the swelling–deswelling processes performed on such types of genipin-crosslinked systems.

### 2.5. Cell Viability Test

**Figure 6:**
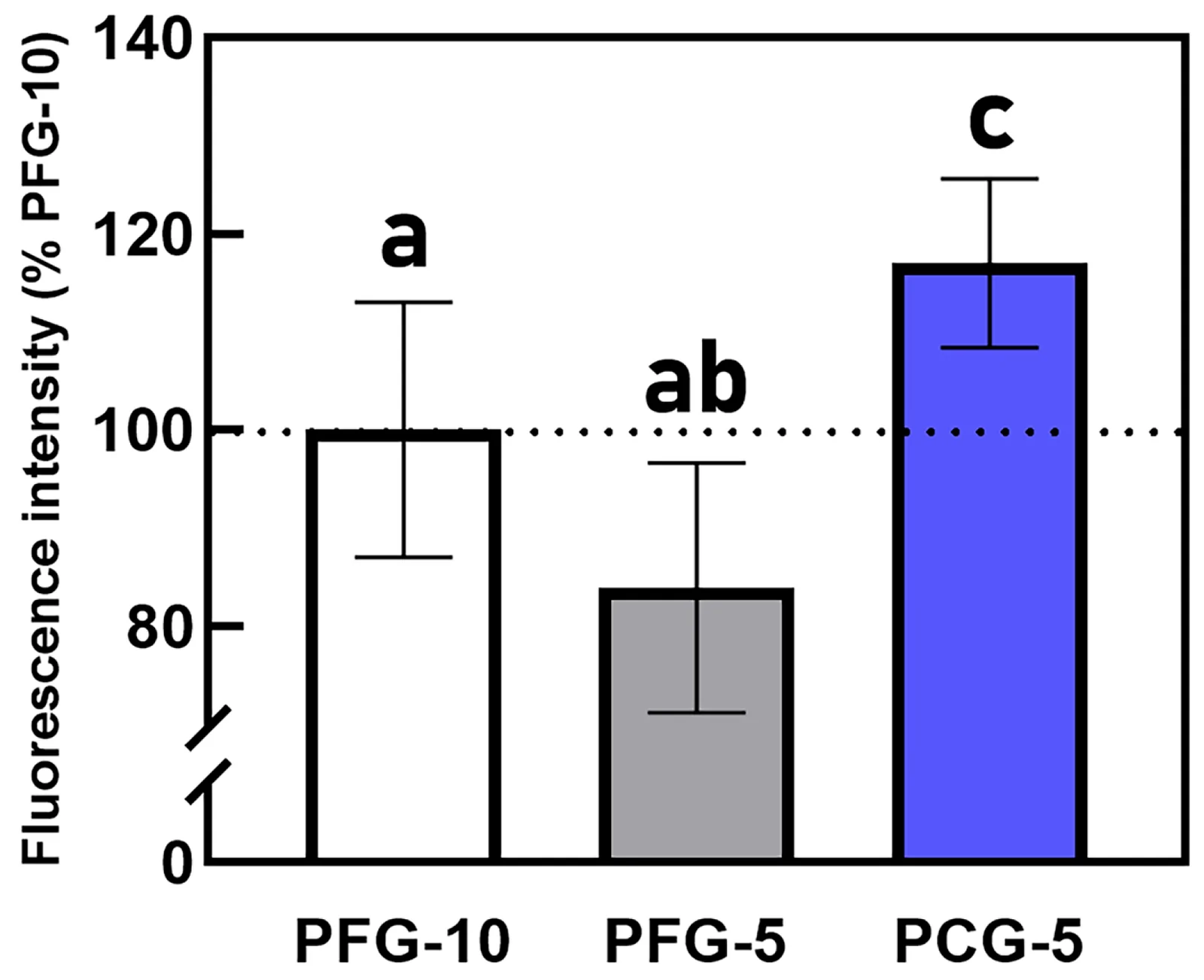
Results of the Alamar Blue assay used to evaluate the cell-adhesive properties of chitosan/carrageenan hydrogels in different cell media: a peptide-free gel in regular cell culture media used as a control (PFG-10); a peptide-free gel in reduced-FBS media (PFG-5); a potentially peptide-conjugated gel in reduced-FBS media (PCG-5). The data marked by different letters are significantly different (*p* < 0.05).

Histograms illustrating the results of an Alamar Blue assay performed following a period of QM7 cell cultivation on genipin-crosslinked chitosan/carrageenan hydrogels are depicted in Figure 6. Briefly, resazurin (Alamar Blue) is a non-fluorescent molecule metabolized by living cells into a fluorescent form, detectable when excited using light of an appropriate wavelength.

QM7 adhesion and proliferation were clearly lower on peptide-free gels incubated in reduced-FBS cell media (83.9 ± 12.7%) than on control gels incubated in regular cell media (100 ± 13.0%). However, potentially peptide-enriched gels incubated in reduced-FBS cell media proved to be excellent scaffolds for QM7 cultivation (117.0 ± 8.6%), even superior to the control gel. Given the lack of significant differences between the rhelogical properties of potentially peptide-conjugated gels and those lacking immobilized peptides, the difference in cell adhesion and metabolism was not attributed to hydrogel mechanical properties, but rather to the potential presence of RGE sequence-containing peptides in the *A. platensis* digest, which may promote cell adhesion in a manner similar to RGD-containing peptides.

Such behavior is in line with the results reported elsewhere on a variety of systems [19,23,48,49,50,51,52]. Thus, this aspect can potentially be explained by the ability of materials potentially functionalized with RGE-sequence-containing peptides to lead to greater protein adsorption, to a similar degree as RGD-sequence-containing peptides, with a direct consequence in improving QM7 adhesion. It is possible that peptide-free gels on regular cell culture media adsorb serum proteins important for cell attachment, such as fibronectin, vitronectin [49], albumin and fetuin-A [50] to a lesser degree than possibly peptide-functionalized gels in reduced-FBS cell culture media, the latter leading to an enhanced cell adhesion to the scaffold.

There is somewhat of a precedent for the use of C-phycocyanin-derived peptides as a replacement for FBS in the culture of myoblasts. Namely, Park et al. [51] examined the potential of C-PC as an alternative to FBS in the cultivation of C2C12 cells, a mouse embryonic myoblast line. Using a CCK-8 assay, all experimental groups containing C-PC showed a higher degree of cell proliferation than groups cultivated in reduced-FBS media not supplemented with C-PC. Further, Dev et al. [52] examined the use of C-PC-conjugated carrageenan hydrogels in wound recovery. The authors assessed the adhesion, proliferation and cell viability of L929 fibroblasts on gels made of 0.5% κ-carrageenan and 0.5% C-PC (*w*/*w*). Although the initial growth rate was slow, it was found that the cells were able to colonize the gel in 5–7 days, their viability exceeded 95% throughout the incubation period and the authors concluded that these gels were adequate matrices for L929 cell cultivation [52].

Additionally, the ability of a *Galdieria sulpharia* extract heat treated at 65 °C for 20–40 min to serve as a replacement for FBS in muscle cell cultivation was examined by Hütker et al. [19] C2C12 cells were cultivated while gradually reducing FBS concentration and gradually increasing extract concentration—cell viability remained unaffected until FBS concentration was reduced below 2%. Glucose consumption and lactate production revealed stable metabolic activity in cultures where FBS was gradually replaced by an algal extract (from 10% FBS to 0% FBS) after an initial adaptation phase. This indicates that these myoblasts can adjust to reduced-FBS conditions or even FBS-free conditions if cell media is supplemented with a *G. sulpharia* extract [19].

Further, the influence of peptides from *Arthrospira platensis* on the proliferation of human amniotic mesenchymal stem cells has previously been studied, albeit in cell media containing 10% FBS. An MTT test was used to assess the metabolic activity of these cells after 48 and 72 h of treatment: it was found that a concentration of certain *Arthrospira platensis* peptides of 300 μg/mL had no cytotoxic effect [49].

Finally, in a study by Mantz et al. [23], the ability of NIH/3T3 fibroblasts to adhere to poly(acrylic acid) (PAA) brushes attached to titanium and interact with DNA complexes on their surface was examined using calcein cell staining and imaging using fluorescent microscopy 48 h after cell seeding. It was found that these fibroblasts adhered to RGD and RGE sequence-containing peptide-conjugated PAA brushes significantly more than non-modified PAA brushes or titanium alone, but no statistically significant difference was observed between RGD- and RGE-modified brushes.

Based on these findings, it is not surprising that algae-derived peptides could perform some of the same roles of FBS, such as promoting cell attachment, while not negatively affecting cell proliferation and viability.

### 2.6. Live/Dead Assay

**Figure 7:**
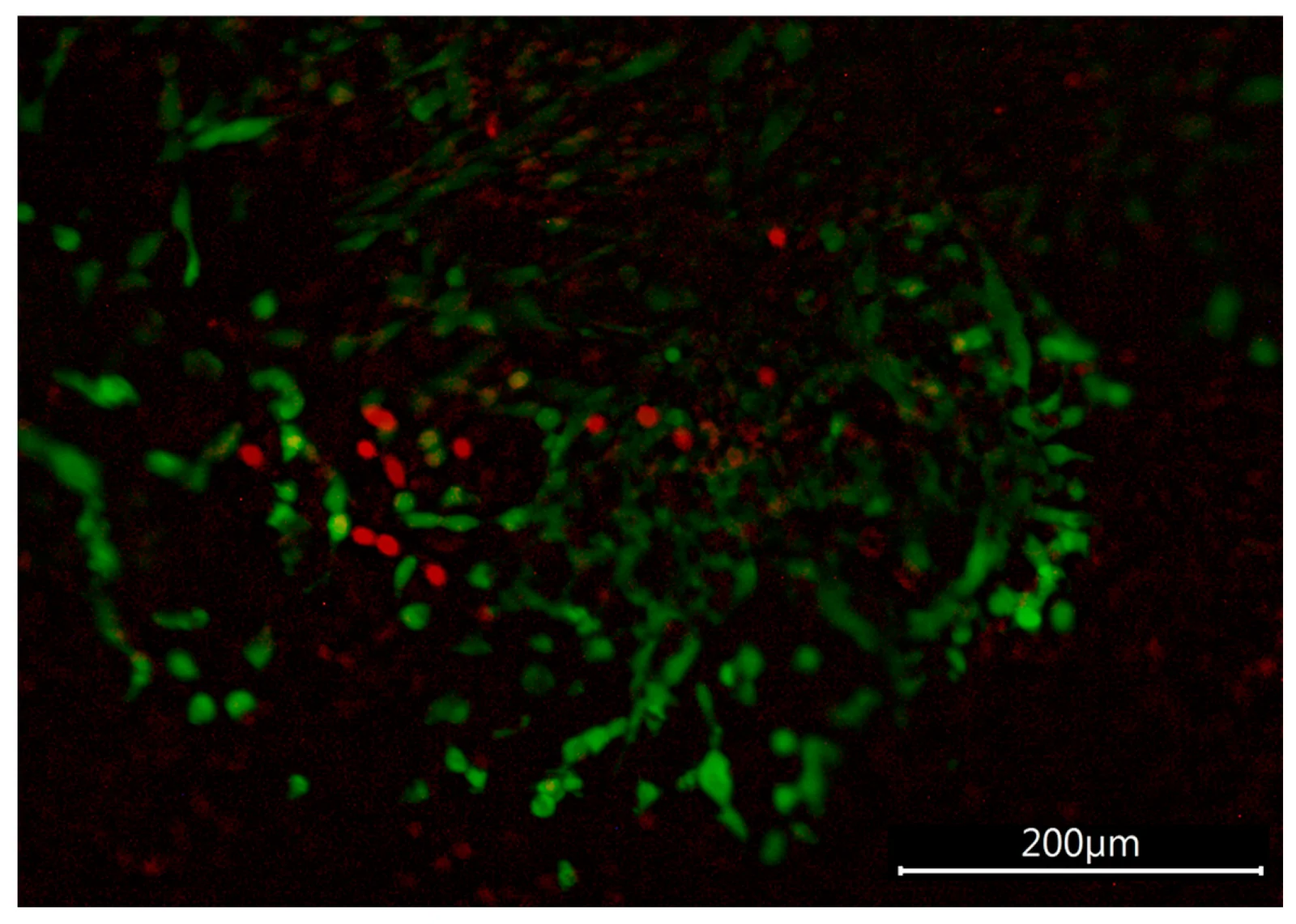
Results of the QM7 cell live/dead imaging assay done on a potentially peptide-conjugated gel incubated in reduced-FBS cell media after three weeks, overlaid.

The results of the live/dead staining assay are shown in Figure 7. Briefly, live cells take up non-fluorescent calcein acetoxymethyl and convert it into fluorescent calcein, which remains trapped inside these cells and has emits green light when excited using light of an appropriate wavelength. Dead cells do not metabolize this compound. Conversely, only dead cells take up ethidium homodimer-1, which binds to DNA, upon which it emits red light if excited using light of an appropriate wavelength.

The image of the dead QM7 cells had a high background signal, likely due to the genipin moieties of the hydrogels. It has been reported that genipin-crosslinked collagen hydrogels are fluorescent, with the emission maximum highly dependent on the excitation wavelength: Hwang et al. [53] studied the increase in fluorescence intensity of a collagen hydrogel during the process of crosslinking using genipin. Fluorescence grew in intensity for twenty-four hours, at which point it was approximately 150,000 times stronger than initially. What is more, this phenomenon is not limited to collagen hydrogels but applies to chitosan hydrogels too: the decrease in intrinsic fluorescence of genipin-crosslinked chitosan/PEG hydrogels has been used by Vo et al. [54] to study their in vitro degradation by lysozyme. In both studies, the excitation wavelength used was around 580–590 nm and the emission maximum was 630 nm. Therefore, the high background fluorescence in the red range is yet another confirmation of successful genipin-induced crosslinking.

Based on ImageJ processing and analysis, which involved background subtraction and contrast enhancement to minimize the effect of intrinsic gel fluorescence caused by genipin-induced crosslinking, it was found that live cells, dyed green, take up roughly 10% of the area of the captured image; whereas, dead cells, dyed red, take up roughly 0.4% of the area, meaning the live cells outnumber dead ones approximately 25-fold after three weeks of cultivation on a potentially peptide-enriched chitosan/carrageenan gel in reduced-FBS cell culture media. Although these are approximate values based on image analysis, it can be concluded that potentially C-phycocyanin-derived peptide-conjugated gels are adequate platforms for QM7 cell cultivation in reduced-FBS media as they allow for their attachment and proliferation.

Gao et al. [55] tested the cell adhesion and viability of L929 fibroblasts on chitosan hydrogels made with different concentrations of both chitosan and genipin. The lowest concentration of genipin used was 1.65 mM, which is a significantly greater concentration than that used in the present study. It was noticed that chitosan gels made with 1.5% solutions of chitosan had good attachment behavior, though it was found that greater concentrations of genipin were associated with better cell attachment. Similar results were observed in the cell viability experiment, with higher concentrations of both components being associated with greater cell viability.

In addition to the previously mentioned effects of algal/cyanobacterial proteins and RGE sequence-containing peptides on cell attachment, an important property of C-phycocyanin-derived phycocyanobilin-containing chromopeptides needs to be considered—particularly, their ability to interact with different cations [20,56]. Crespi and Smith [56] demonstrated that C-PC-derived chromopeptides interact with a number of metal ions, such as Hg^2+^, Ag^+^ [20,44], Pb^2+^, Cu^2+^ [20], Cd^2+^, Fe^2+^, Mn^2+^, and Co^2+^ [56]. Similarly, Lin and Metters [57] showed that proteins such as bovine serum albumin adhere to poly(ethylene glycol) hydrogels with immobilized iminodiacetic acid better in the presence of certain metal ions, such as Cu^2+^, Zn^2+^, and Ni^2+^. Considering the importance of serum proteins for cell attachment [38,40], the ability of C-phycocyanin-derived peptides to interact with metal ions present in cell media and FBS must be considered one of the potential ways of promoting protein attachment, and subsequently, cell attachment.

### 2.7. Preliminary Cost Assessment and Study Limitations

A preliminary cost assessment was performed to evaluate the economic feasibility of the proposed hydrogel formulation (Table S1). Direct scaling of the laboratory formulation to 1 kg of freeze-dried hydrogel requires approximately 333 g each of chitosan and carrageenan and 35.6 g of genipin. For peptide-conjugated hydrogels, approximately 3.56 mmol of phycocyanobilin-equivalent peptides would additionally be required, corresponding to approximately 154 g of Blue Spirulina based on the experimentally determined digest yield. Using current laboratory reagent prices, the estimated production cost is approximately €8.8 × 10^3^ kg^−1^, with research-grade genipin representing the dominant cost contributor, while peptide incorporation increases the total cost by only approximately 1%. Under an indicative bulk/pilot-scale scenario, the estimated costs decrease substantially to approximately €80–95 kg^−1^ and €135–155 kg^−1^ for peptide-free and peptide-conjugated hydrogels, respectively. Compared with scaffold functionalization using purified or chemically synthesized cell-adhesive peptides, such as RGD-containing peptides, the use of an unfractionated cyanobacterial digest avoids peptide synthesis and extensive purification, representing a potential economic advantage. Conversely, genipin crosslinking, multiple freeze-drying steps, and the high liquid-to-solid ratio of the present protocol represent important limitations compared with simpler hydrogel fabrication approaches. Thus, future optimization should focus on reducing solvent volumes and lyophilization steps and using bulk-grade materials. These estimates are preliminary and exclude labor, capital, quality-control, and regulatory costs; therefore, a comprehensive techno-economic analysis following process optimization and scale-up will be required to establish the commercial feasibility of the proposed system.

There are several limitations to this study that need to be acknowledged. Firstly, only one cell culture was used. Secondly, excluding peptide immobilization and lack thereof, only one type of gel was used, with no variation in polysaccharide and/or genipin concentrations. Thirdly, an *A. platensis* digest was used, instead of a specific peptide, or a few specific peptides, whose amino acid sequence could be linked to specific biological activities.

## 3. Conclusions

In the present study, novel hydrogels were formulated and fabricated using algal polysaccharide carrageenan, chitosan and *Arthrospira platensis*-derived peptides with the goal of developing a platform for myoblast cultivation in reduced-FBS conditions and without the use of animal-derived biopolymers. These gels were shown to enable QM7 cell cultivation in reduced-FBS conditions for periods of up to three weeks at least, with cell viability comparable to cell viability in cell media containing regular amounts of FBS. Based on the results obtained in the present study, as well as the information gathered in existing literature, it can be concluded that potentially chemically crosslinked, *Arthrospira platensis*-derived peptide-conjugated chitosan/carrageenan hydrogels are potentially a promising platform for long-term myoblast cultivation with minimal use of animal-derived products on a laboratory scale. Further studies need to focus on optimizing the exact formulation of these gels, such as polysaccharide concentration, crosslinking methods, crosslinker concentration, among other factors, to allow further reduction in FBS concentration, greater cell adhesion, proliferation, and viability. Further, additional cell lines should be used to assess the potential of these hydrogels to serve as platforms for their cultivation.

## 4. Materials and Methods

### 4.1. Materials

Food-grade *Arthrospira platensis* powder was purchased from We Are One, Rade Končara 10, Sombor, Serbia. Anhydrous dibasic sodium phosphate (71640-1KG, p.a.), calcium chloride (223506-500G), chitosan (low molecular weight, 448869-50G), carrageenan (suitable for gel preparation, C1013-25G), tryptose phosphate broth (T8159-100ML, sterile-filtered and suitable for cell culture), and resazurin sodium salt (R7017-1G) were purchased from Sigma-Aldrich, Co. (3050 Spruce Street, St. Louis, MO 63103, USA). Ammonium sulfate (Art.-Nr.3746.3, ≥99.5%, p.a.) and chymotrypsin (Art.-Nr.0238.9, ≥1 000 USP-U/mg, for biochemistry) were purchased from Carl Roth GmbH+Co. KG Schoemperlenstrasse. 3-5—D-76185 Karlsruhe, BW, Germany. Bidistilled glycerol was purchased from VWR Chemicals, part of Avantor, One Radnor Corporate Center, Building One, Suite 200, 100 Matsonford Road, Radnor, PA 19087, USA. Sephadex-G25 (17003201) was purchased from Cytiva, 100 Results Way Marlborough, MA, 01752-3078 USA. Acetic acid (607-002-00-6, p.a.) was purchased from Centrohem, Vuka Karadžića bb 22300, Stara Pazova, Serbia. Genipin (BD131935-1G) was purchased from BLD Pharmatech Ltd., 488 Taoqiao Road, Building 6, 1F, HuiNan Town, Pudong New Area, Shanghai 201399, China. Absolute ethanol (E/0665DF/17, HPLC grade) was purchased from Fisher Scientific, Bishop Meadow Road, Loughborough, LE11 5RG, UK. Gibco 1X M199 cell culture media (11043-023), fetal bovine serum (A5256701) and a LIVE/DEAD™ Viability/Cytotoxicity Kit (L3224) were purchased from Thermo-Fisher Scientific, 168 Third Ave, Waltham, MA 02451, USA. QM7 cells were purchased from the American Type Culture Collection (10801 University Boulevard, Manassas, VR 20110, USA).

### 4.2. Arthrospira Platensis Extract and Enzymatic Digest Preparation

An *Arthrospira platensis* extract was prepared using an *Arthrospira platensis* powder (We Are One, Rade Končara 10, Sombor, Serbia) and a 20 mM phosphate-buffered solution (pH 7.2, MiliQ water) (3050 Spruce Street, St. Louis, MO 63103, USA)—20 mL of buffer solution was used for each gram of dry *Arthrospira platensis* powder. The extraction was done at room temperature for 4 h with constant stirring using a magnetic stirrer. The raw extract was subsequently centrifuged at 10,000 *g* for 20 min at 4 °C (Heraeus Biofuge Primo R, Thermo-Fisher Scientific, 168 Third Ave, Waltham, MA 02451, USA): the sediment was discarded, and the volume of the supernatant was measured. Powdered ammonium sulfate (Carl Roth GmbH+Co. KG Schoemperlenstrasse. 3-5—D-76185 Karlsruhe, BW, Germany) was added to the supernatant with constant stirring by a magnetic stirrer to a concentration of 65%. This solution was left at 4 °C overnight and subsequently centrifuged at 10,000 *g* for 20 min at 4 °C. The supernatant was discarded and the sediment was resuspended in a minimal volume of 20 mM phosphate-buffered solution (pH 7.2, MiliQ water). This solution was then dialyzed overnight against a 20 mM phosphate-buffered solution (pH 7.2). The dialyzed extract was then centrifuged at 10,000 *g* for 20 min at 4 °C, and the sediment was discarded. The volume of the supernatant was determined, and bidistilled glycerol (VWR Chemicals, part of Avantor, One Radnor Corporate Center, Building One, Suite 200, 100 Matsonford Road, Radnor, PA 19087, USA) was added to it, to a concentration of 33% (*v*/*v*). The concentrations of C-phycocyanin (C-PC) and allophycocyanin (A-PC), were calculated using the following equations [58]:

$$\left[C-PC\right]\;\left(\frac{mg}{mL}\right)\;=\;\frac{A_{618}\;-\;\left(0.474\;\frac{mg}{mL}\;\times \;A_{652}\right)}{5.34}$$

$$\left[A-PC\right]\;\left(\frac{mg}{mL}\right)\;=\;\frac{A_{652}\;-\;\left(0.205\;\frac{mg}{mL}\;\times \;A_{618}\right)}{5.09}$$

The absorbance values in the above equations should be in the range of 0.05–1.00. The extract thus obtained was used to prepare an enzymatic digest using chymotrypsin (Carl Roth GmbH+Co. KG Schoemperlenstrasse. 3-5—D-76185 Karlsruhe, BW, Germany). 0.5 IU of chymotrypsin were used for each gram of phycobiliprotein in the extract, which was diluted 5 times using 20 mM phosphate-buffered solution (pH 8, MiliQ water). Subsequently, a calcium chloride solution (3050 Spruce Street, St. Louis, MO 63103, USA) was added to the digestion mixture to a concentration of 1 mM. The digestion mixture was left overnight at 37 °C inside an Orbital Shaker-Incubator ES-20 (SIA Biosan, Ratsupites iela 7 k-2, Riga, Latvia, LV-1067). The mixture was then centrifuged at 10,000 *g* for 20 min at 4 °C, and the sediment was discarded. The rest of the supernatant was gel-filtered using a Sephadex-G25 Fine (Cytiva, 100 Results Way Marlborough, MA, 01752-3078 USA) column in order to separate peptides from undigested PBPs; the digest was eluted using a 5 mM phosphate buffer (pH 7.2, MiliQ water), which the matrix was also kept in. Once the peptides had been collected, the digest was again centrifuged at 10,000 *g* for 20 min at 4 °C, the supernatant was frozen at −70 °C, and freeze dried for 24 h using an Alpha 2–4 LD Plus freeze-dryer (Martin Christ Gefriertrocknungsanlagen GmbH, An der Unteren Soese 50, 37520 Osterode am Harz, NI, Germany), and then the dry remaining mass was dissolved in a minimal volume of MiliQ water to obtain the final digest. The digest concentration, expressed as phycocyanobilin chromophore equivalent, was determined using a standard curve constructed using the aliquot taken before the gel-filtration step as described in Gligorijević et al. with slight modifications [59].

### 4.3. Hydrogel Preparation and Peptide Conjugation Assay

Chitosan/carrageenan-based hydrogels were prepared by a suspension-casting method. A 3% (*w*/*v*) solution of chitosan (3050 Spruce Street, St. Louis, MO 63103, USA) was prepared by dissolving chitosan in 0.1 M acetic acid (Centrohem, Vuka Karadžića bb 22300, Stara Pazova, Serbia) (MiliQ water) with constant stirring using a magnetic stirrer. A 3% (*w*/*v*) carrageenan (3050 Spruce Street, St. Louis, MO 63103, USA) suspension was prepared by suspending carrageenan in a 50 mM cell-grade phosphate buffer (pH 7.2, MiliQ water) with constant stirring using a magnetic stirrer. According to the manufacturer, this carrageenan contains <3.5% Ca^2+^, <11% K^+^ and <2% Na^+^ (*w*/*w*). Subsequently, the chitosan solution was added to the carrageenan suspension with constant stirring using a magnetic stirrer in a 1:1 (*v*/*v*) ratio. Once an apparently homogeneous dispersed mixture of these polysaccharides was obtained, it was cast into 6- and 48-well plates (Sarstedt AG & Co. KG, Sarstedtstrasse 1, D-51588, Nuembrecht, NW, Germany), which served as molds. Approximately 500 μL was cast into the wells of the 48-well plate, and approximately 1 mL was added to the wells of the 6-well plate. Once frozen at −70 °C, these mixtures were freeze-dried for 24 h. Some freeze-dried mixtures were set aside and not treated with genipin; they were stored at −20 °C in parafilm-sealed vials until further use. Two gelation solutions were prepared. The first was used to obtain genipin-linked chitosan/carrageenan hydrogels without immobilized peptides: genipin (BLD Pharmatech Ltd., 488 Taoqiao Road, Building 6, 1F, HuiNan Town, Pudong New Area, Shanghai 201399, China) was dissolved in a cell-grade 50 mM phosphate buffer (pH 6.5, MiliQ water) to obtain a 0.1% (*w*/*v*) genipin solution. The second solution was prepared the same way, except a chymotrypsin digest of a *Arthrospira platensis* was added at a chromophore concentration of 100 μM. These solutions were left at room temperature for 30 min before being added to the freeze-dried mixtures of chitosan/carrageenan. An amount of 800 μL of these solutions was added to the wells of the 48-well plate and 3 mL was added to the wells of the 6-well plate. The plates were left at room temperature for 24 h to allow complete crosslinking. Subsequently, the genipin linked chitosan/carrageenan hydrogels were frozen at −70 °C, freeze-dried for 24 h, and stored at −20 °C in parafilm sealed vials until further use.

Spectrophotometry was used to estimate *A. platensis* peptide conjugation had taken place. Two solutions were prepared—a 100 μM *A. platensis*-chymotrypsin digest in a 50 mM phosphate buffer (pH 7.2) but no genipin, and a 100 μM *A. platensis*-chymotrypsin digest in the same buffer and 0.1% (*w*/*v*) genipin. Non-crosslinked chitosan/carrageenan gels were weighed to ensure complexes of comparable masses (average weight: 21.35 mg; standard deviation: 1.65) were used and were subsequently placed in the wells of a 48-well plate. Half of these gels were submerged in 800 μL of the genipin-free solution, and the other half were submerged in 800 μL of the solution containing genipin. In addition, the genipin-free and genipin-containing solutions were added to wells without gels. The plate was left at room temperature for 24 h. A 50 mM phosphate buffer (pH 7.2) was used as a blank. Subsequently, the absorbance of every solution at 581 nm (A_581_) was measured. The percentage of peptides likely covalently bound to the chitosan/carrageenan gel was calculated using the following equations:

% of peptides immobilized (no genipin) = (1 − (A_CDG_/A_0DG_)) × 100

% of peptides immobilized (with genipin) = (1 − (A_CD_/A_0D_)) × 100

where A_CDG_ is the A_581_ of the digest- and genipin-containing solutions in the wells containing gels, A_0DG_ is the average A_581_ of the digest- and genipin-containing solutions in the wells without gels, A_CD_ is the A_581_ of the genipin-free digest-containing solutions in the wells containing gels, and A_0D_ is the average A_581_ of the genipin-free digest-containing solutions in the wells without gels. The results of this assay are shown in the Supplementary Materials.

### 4.4. Scanning Electron Microscopy (SEM)

Genipin-crosslinked chitosan/carrageenan hydrogel and non-crosslinked mixture microstructures were examined by scanning electron microscopy. The microscope used was a JSM–6390LV (JEOL, 3-1-2 Musashino, Akishima, Tokyo 196-8558, Japan). The freeze-dried samples prepared as described in Section 4.3. *Hydrogel Preparation* were kept at room temperature for thermal equilibration and coated with a thin layer of gold prior to being exposed to the electron beam of microscope. The SEM images of their surfaces were captured at an accelerating voltage of 20 kV, with magnifications of ×200 and ×600.

ImageJ was used to estimate the number of pores, average pore size, and the percentage of the area taken up by pores in SEM images at magnification ×200. The scale bar present in the images was used to set the scale used by the software. All the area above the scale bar was used for image analysis. The image threshold was adjusted to highlight pores and exclude the area between them, whereupon the resulting particles were analyzed by excluding those with a negligibly small area to reduce noise.

### 4.5. Fourier-Transform Infrared Spectroscopy

For FTIR analysis, freeze-dried genipin-crosslinked chitosan/carrageenan hydrogel samples, a non-crosslinked chitosan/carrageenan mixture, and chitosan and carrageenan separately, all prepared in 48-well plates, were homogenized with potassium bromide (KBr) and compressed into pellets using a hydraulic press. The spectra were recorded at room temperature in the spectral range of 400–4000 cm^−1^ using an IRAfinity-1 FTIR spectrometer (Shimadzu, 1, Nishinokyo Kuwabara-cho, Nakagyo-ku, Kyoto 604-8511, Japan), with the total number of accumulations per sample being 100 at a resolution of 4 cm^−1^.

### 4.6. Rheological Property Analysis

The rheological properties of a non-crosslinked chitosan/carrageenan mixture, a peptide-free genipin-crosslinked chitosan/carrageenan hydrogel, and a potentially peptide-conjugated genipin-crosslinked, all prepared in 6-well plates as described in Section 4.3 *Hydrogel preparation*, were determined. All the samples were disk shaped. A Discovery Hybrid Rheometer HR2 (TA Instruments, 159 Lukens Drive, New Castle, DE 19720, USA) in parallel plate geometry was used. The upper plate of the device had a diameter of 25 mm, and the lower plate was a stainless-steel plate whose temperature was controlled using a Peltier device. Oscillatory sweep tests were performed with varying frequencies from 0.1 to 100 rad/s, with a strain amplitude of 1% ascertained to be in the linear viscoelastic range, at 25 °C.

### 4.7. Water Uptake and Retention Capacity

The water uptake capacity of genipin-crosslinked chitosan/carrageenan gels with and without potentially immobilized C-phycocyanin-derived peptides prepared using a 48-well plate as a mold was determined using the following method. Freeze-dried gels were measured prior to the experiment using an ATX224R analytical balance (Shimadzu, 1, Nishinokyo Kuwabara-cho, Nakagyo-ku, Kyoto 604-8511, Japan), and then left to swell in de-ionized water, and their mass was measured after 30 min, 1, 1.5, 2, 3, 4, 6, 8, 24, and 48 h. The water uptake capacity of the gels at different times was calculated using the following equation:

Water Uptake Capacity (%) = (W_t_ − W_0_)/W_0_ × 100%

where W_t_ is the mass of the sample at time t, and W_0_ is the mass of the sample before the start of the experiment.

The water retention capacity of genipin-linked chitosan/carrageenan gels with and without potentially immobilized C-phycocyanin-derived peptides prepared using a 48-well plate as a mold was determined by the following method. The mass of the gels was determined using an analytical balance, then left at room temperature in a sealed plastic container along with wet tissues to create a humid atmosphere. Their mass was then measured after 30 min, 1, 1.5, 2, 3, 4, 6, 8, 24 and 48 h. The water-retention capacity of the gels at different times was calculated using the following equation:

Water Retention Ratio (%) = W_t_/W_0_ × 100%

where W_t_ is the mass of the sample at time t, and W_0_ is the mass of the swollen sample before the start of the experiment.

### 4.8. Cell Viability Test

Freeze-dried genipin-crosslinked chitosan/carrageenan gels with and without potentially immobilized C-phycocyanin-derived peptides prepared using a 48-well plate as a mold were sterilized by submerging them in a 70% (*v*/*v*) absolute ethanol-MiliQ water solution for an hour. Subsequently, these were transferred to a 48-well plate for cell suspensions (Sarstedt AG & Co. KG, Sarstedtstrasse 1, D-51588, Nuembrecht, NW, Germany) inside a laminar flow hood and each washed three times for 30 min using 500 μL of sterile phosphate-buffered saline each time, after which the gels were divided into three groups. Nine gels not enriched with C-phycocyanin-derived peptides were cultivated in M199 cell culture media (Thermo-Fisher Scientific, 168 Third Ave, Waltham, MA 02451, USA) containing a 1% antibiotic mixture (penicillin, streptomycin), 10% sterile-filtered tryptose phosphate broth (3050 Spruce Street, St. Louis, MO 63103, USA) suitable for cell culture and 10% FBS (Thermo-Fisher Scientific, 168 Third Ave, Waltham, MA 02451, USA). Nine of the gels potentially enriched with C-phycocyanin-derived peptides were also incubated in the same cell media. The other nine were incubated in the same cell growth media, except the FBS concentration was reduced to 5%. QM7 cells (American Type Culture Collection, 10801 University Boulevard, Manassas, VR 20110, USA) were then seeded: 500,000 cells, at a seeding density of 5,000,000 CFU/mL, were pipetted onto the gels. Once the gels had absorbed the cell suspension, 400 μL of M199 cell media was added, with the same FBS concentration as before. Corresponding gels, onto which cells were not seeded, were incubated in the same conditions to serve as control groups. QM7 cells were cultured in these conditions for 7 days.

Following the 7-day culture period, an Alamar Blue assay was used to assess the cell viability of the four QM7 cell populations. A stock solution of a resazurin sodium salt (3050 Spruce Street, St. Louis, MO 63103, USA) was diluted in FBS-free M199 cell media according to the manufacturer’s instructions to prepare a working solution of Alamar Blue. Briefly, the gels used as a platform for QM7 cell growth and appropriate control gels were transferred to a new 48-well plate, washed thoroughly with PBS to remove all FBS, after which 500 μL of the working Alamar Blue solution was added to each gel. The gels were incubated at 37 °C for 4 h, after which the solutions were transferred to fluorescence-compatible 96-well plates (Sarstedt AG & Co. KG, Sarstedtstrasse 1, D-51588, Nuembrecht, NW, Germany) and their fluorescence intensity was determined using a BioTek multimode plate reader (Agilent Technologies Inc., 5301 Stevens Creek Boulevard, Santa Clara, CA 95051, USA) with a filter configured at 530/25 nm for excitation and 590/35 nm for emission. The fluorescence of the control gels was subtracted from the fluorescence of the gels used to cultivate QM7 cells.

### 4.9. Live/Dead Assay

QM7 cells were cultured for twenty-one days on a genipin-crosslinked chitosan/carrageenan hydrogel potentially enriched with C-phycocyanin-derived peptides in reduced-FBS cell culture media. Subsequently, a live/dead staining assay was performed according to the manufacturer’s instructions to visually confirm the presence of viable cells on the gel. Briefly, the gel was thoroughly washed with sterile PBS. A working solution of 2 μM calcein acetoxymethyl (Thermo-Fisher Scientific, 168 Third Ave, Waltham, MA 02451, USA) and 4 μM ethidium homodimer-1 (Thermo-Fisher Scientific, 168 Third Ave, Waltham, MA 02451, USA) solution was prepared in PBS and then the hydrogel was incubated in the dye solution at room temperature for approximately 30 min. Subsequently, it was imaged using an A16.2701 fluorescent microscope (Opto-Edu, F-1501 Wanda Plaza, No.18 Shijingshan Road, Beijing 100043, China) equipped with filters with excitation/emission maxima at 465–495 nm/515–555 nm and 521.5–552.5 nm/~590 nm to observe live and dead cells, respectively. The images were captured using ImageView (BestScope, 1201-3, Block B, Zhonghai Plaza, No. 255 Chengxing Street, Shijingshan District, Beijing, China), processed and analyzed using ImageJ (version 1.54g; National Institute of Health, Bethesda, USA) and overlaid using GIMP (GNU Image Manipulation Program version 3.2.4). The images were processed in ImageJ by subtracting the background and enhancing the contrast to lower background noise and then analyzed by converting them into 8-bit and then using the Analyze Particles tool. The processed images were overlaid.

### 4.10. Statistical Analysis

The results of the Alamar Blue cell viability assay were analyzed using a one-way ANOVA and a post hoc Tukey test. The results of the peptide immobilization assay were analyzed using a *t*-test. All experiments were done at least in triplicate.

## Data Availability

Additional data will be made available upon request.

## Supplementary Materials

The following supporting information can be downloaded at: https://www.mdpi.com/article/10.3390/gels12080720/s1, Figure S1. Results of the peptide conjugation assay; Figure S2. FTIR spectra of chitosan and carrageenan; Table S1. Preliminary Cost Assessment for 1 kg of Freeze-Dried Hydrogel.

## Abbreviations

The following abbreviations are used in this manuscript:

ANOVA	Analysis of variance
FBS	Fetal bovine serum
CPC	C-phycocyanin
APC	Allophycocyanin
SEM	Scanning electron microscopy
PEG	Poly(ethylene glyocol)
MA	Methyl methacrylate
AEMA	2-aminoethyl methacrylate hydrochloride
mPEG-MA	Methyl ether methacrylate methoxy poly(ethylene) glycol
MTT	3-(4,5-di methyl thiazol-2-yl)-2,5-diphenyltetrazolium bromide
CM	Cultivated meat
PEC	Polyelectrolyte complex
ECM	Extracellular matrix
TFF	Tangential flow filtration
HPLC	High-performance liquid chromatography
p.a.	pro analysi
FTIR	Fourier-transform infrared spectroscopy
CFU	Colony-forming units
PBS	Phosphate-buffered saline
QM7	Quail muscle clone 7
GIMP	GNU Image Manipulation Program
