# Supplementary material for: C-Phycocyanin-Derived Peptide-Conjugated, Chemically Crosslinked Chitosan/Carrageenan Hydrogels for Myoblast Cultivation in Reduced Serum Conditions
Source: Gels. 2026 Aug 13;12(8):720. doi: 10.3390/gels12080720 (PMC13511876; doi:10.3390/gels12080720)
Supplement: Supplementary file 1 [file gels-12-00720-s001.zip › gels-4445692-supplementary.pdf]

SUPPLEMENTARY MATERIAL

FOR

C-Phycocyanin-Derived Peptide-Conjugated, Chemically Crosslinked Chitosan/Carrageenan Hydrogels for Myoblast Cultivation in Reduced Serum Conditions

Ljubodrag Aleksić[1], Vladimir Pavlović[2], Marija S. Nikolić[3], Aleksandar Ivanov[1], Nikola Gligorijević[4] and Simeon Minić[1,*]

[1]University of Belgrade – Faculty of Chemistry, Centre of Excellence for Molecular Food Sciences & Department of Biochemistry, Belgrade, Serbia

[2]University of Belgrade – Faculty of Agriculture, Department of Mathematics and Physics, Belgrade, Serbia

[3]University of Belgrade – Faculty of Technology and Metallurgy, Department of General and Inorganic Chemistry, Belgrade, Serbia

[4]University of Belgrade, Institute of Chemistry, Technology, and Metallurgy, National Institute of the Republic of Serbia, Department of Chemistry, Belgrade, Serbia

*Correspondence: sminic@chem.bg.ac.rs

1.Results

1.1 Peptide Conjugation Assay

The results of the peptide conjugation assay are depicted in Figure 1.

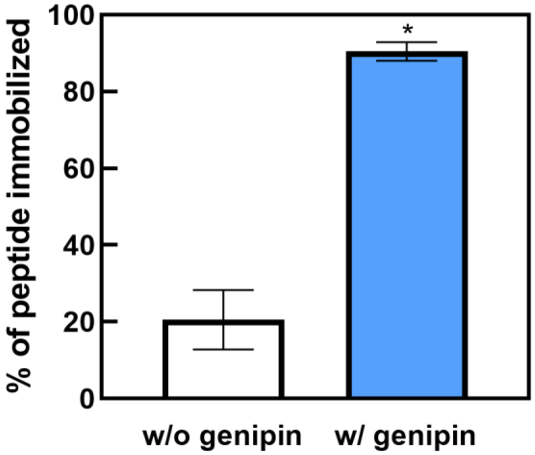

Figure S1. Results of the peptide conjugation assay. **W/o genipin** represents the percentage of peptides immobilized in wells containing a genipin-free 100 µM *A. platensis*-chymotrypsin digest and a chitosan/carrageenan mixture and **w/ genipin** represents the percentage of peptides immobilized in wells containing a 100 µM *A. platensis*-chymotrypsin digest containing 0.1% genipin (*w/v*) and a chitosan/carrageenan mixture. * denotes significant difference with $p < 0.0001$

1.2 Chitosan and Carrageenan FTIR Spectra

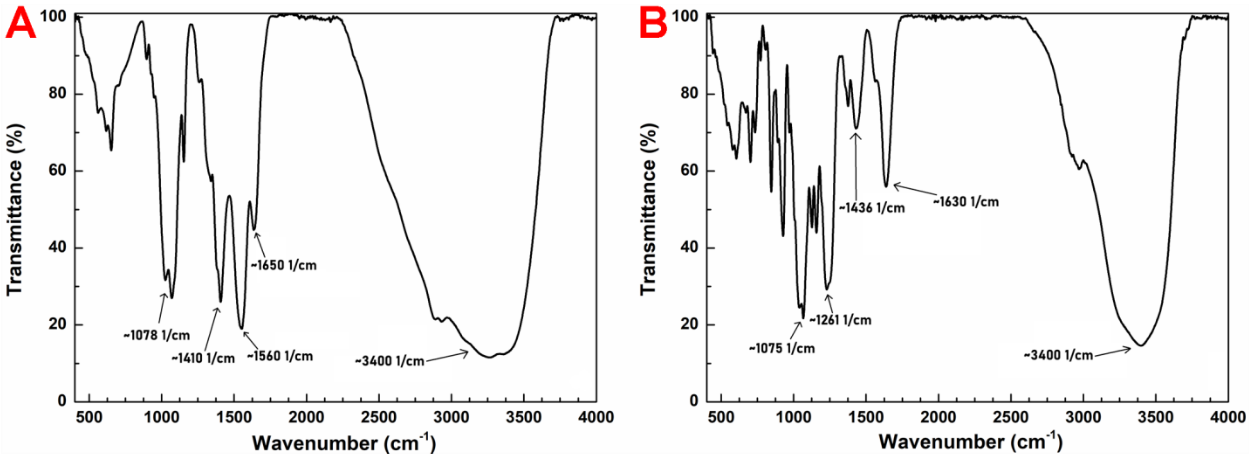

Figure S2. FTIR spectra of chitosan (**A**) and carrageenan (**B**).

1.3. Cost Estimation

Laboratory-scale material costs were estimated using publicly available retail prices of research- or laboratory-grade reagents from commercial suppliers, preferentially using the same suppliers or

equivalent grades as those employed experimentally. Prices for chitosan and carrageenan were based on Sigma-Aldrich/Merck catalogue prices, genipin on commercial research-grade supplier pricing, and chymotrypsin on the Carl Roth catalogue price. The cost of Blue Spirulina was estimated from current European retail prices for commercially available Blue Spirulina powder. For the indicative pilot/industrial-scale scenario, bulk-market prices for food/technical-grade chitosan, carrageenan, genipin and Blue Spirulina were used instead of research-grade catalogue prices. Buffer components, acetic acid, salts and process water were estimated from bulk chemical prices, while freeze-drying costs were approximated from the processing volume, equipment capacity and electricity requirements. All prices were converted to EUR and normalized to the quantities required to produce 1 kg of freeze-dried hydrogel. Because bulk prices vary substantially with supplier, purity, order volume and geographical region, the pilot-scale values should be regarded as indicative estimates rather than fixed production costs. Prices were accessed in August 2026.

Table S1. Preliminary Cost Assessment for 1 kg of Freeze-Dried Hydrogel

Component / operation	Amount per 1 kg dry-gel equivalent	Laboratory-scale estimate	Pilot/industrial-scale estimate
Chitosan	333.3 g	~€672	~€21–25
Carrageenan	333.3 g	~€150	~€4–6
Genipin	35.56 g	~€7,881	~€25–32
Blue Spirulina (peptide preparation only)	154.1 g	~€54	~€43
Chymotrypsin	~0.023 mg	<€0.01	<€0.01
Buffers, acetic acid, and salts	—	~€25	~€8
Lyophilization / process energy	—	~€80–115	~€32–50
**Hydrogel WITHOUT peptides**	**—**	**≈€8,780/kg**	**≈€80–95/kg**
**Hydrogel WITH peptides**	**—**	**≈€8,860–8,890/kg**	**≈€135–155/kg**
**Increment due to peptide functionalization**	**—**	**≈€80–110/kg (~1%)**	**≈€50–60/kg (~55–70%)**

Note: Values represent preliminary indicative estimates based on direct scaling of the laboratory formulation. Labor, capital expenditure, quality-control, regulatory, and distribution costs are not included.
